# Supplementary material for: Granger sensori-behavioral functional taxonomy of neuronal ensemble activity from two-photon calcium imaging data
Source: PLoS Comput Biol. 2026 Sep 30;22(9):e1014820. doi: 10.1371/journal.pcbi.1014820 (PMC13660992; doi:10.1371/journal.pcbi.1014820)
Supplement: Supplementary file 1 [file pcbi.1014820.s002.pdf]

Response to the Reviewer Comments on "Granger Sensori-Behavioral Taxonomy of Neuronal Ensemble Activity from Two-Photon Calcium Imaging Data" (PCOMPBIOL-D-26-00488)

Sahar Khosravi, Nikolas A. Francis, Patrick O. Kanold, and Behtash Babadi

Preface

This is the revised version of our manuscript titled "Granger Sensori-Behavioral Functional Taxonomy of Neuronal Ensemble Activity from Two-Photon Calcium Imaging Data" (PCOMPBIOL-D-26-00488). Thanks to the comments and feedback of the reviewers, we have now substantially revised our manuscript by including several new analyses, clarifying our methodological contributions and their relation to existing work, and improving the clarity and rigor of our expositions. In summary, the major changes include:

1) Adding several new analyses to assess the robustness of our proposed method to more realistic experimental conditions (Fig 3 and pages 13-16);
2) Including several new subsections in the Methods to rigorously evaluate the computational complexity of our method as well as existing ones, describing the details of the benchmark methods and data pre-processing (pages 43-45);
3) Adding several new discussion points regarding the relation of our methodological contributions to existing work such as CCM and cNMF, and also discussing the limitations and caveats of our proposed G-taxonomy in more details (pages 24-27); and
4) Clarifying how are results confirm existing literature and how they provide new insights (pages 20-22).

In what follows, we respond (in black) to the comments of the reviewers (in green) in a point-by-point fashion.

We have included an identical version of the manuscript that highlights all the changes in blue for ease of tracking. We would like to thank the reviewing editor and the anonymous reviewer for their careful critique of our work and for their constructive and thorough feedback.

Response to Editor's Comments

The three reviewers are positive about the approach developed in the paper. However they have also made extensive comments and suggestions that need addressing for the paper to meet publication requirements.

Response: We thank the editor for handling the review process and for summarizing the outcome. We have now constructively and thoroughly incorporated all the reviewer comments, which has significantly enhanced the quality of our work.

Response to Reviewer 1's Comments

Reviewer #1: The manuscript by Khostavi et al., entitled "Granger Sensori-Behavioural Taxonomy of Neuronal Ensemble Activity from Two-Photon Calcium Imaging Data", immediately stands out as a well-thought-out and executed piece of work that improves our understanding of neuronal computation. It sets out to build and test a computational model that provides an all-in-one account of sensory processing. By all-in-one, they mean both the encoding and decoding of stimuli by neurons, among other things, which together constitute the neuronal response. Therefore, it is laudable that the authors took an approach that is contrary to the previous approach, which slices up neural activity in the study of one or the other, i.e., encoding or decoding of sensory information. With the latter approach, recent developments in statistical hypothesis testing have shown that type I error, thus false positives, abound.

The authors' model is a classification system that describes three categories of neurons related to sensory and behavioural information in neural ensemble activity, based on a correlational metric known as Granger formalism. To be able to infer information, in the spirit of existing frameworks like intersection information and partial information decomposition, Granger causality (GC) presents a robust parametric tool to identify directional effects and within multivariate point process data. To this end, the three categories of neurons were defined as Granger stimulus encoding (GS), Granger behavioural decoding (GB), and Granger Intersection (GI), guided by efficient statistical testing, control of false discovery rate, and effective estimation of network parameters.

To test the model, the authors sought to identify GS, GB, and GI in two-photon calcium imaging data. Further, the marked neurons were hypothesised to approximate neurons in the behaving mouse A1, with similar functions. The results showed a significant presence of GC neurons in both datasets.

Response: We thank the reviewer for their positive assessment of our work.

While the authors presented good evidence to support the model, there remain significant points that allow for confidence in the utility of the model to be shaken, especially for experimenters as well as theoreticians. These points can be put into two folds: manifested or assumed properties of neurons ascribed to GC neurons and the physical connectivity of identified properties between GC neurons. By their nature, models are physical description of the constitution and mechanism of systems. Hence, the authors' model lacks a cortical structure and precise transduction of information between GC neurons, which the statistical framework, Granger formalism, was supposed to elucidate. For instance, GS, GB, and GI neurons were shown to characterise diverse groups of neurons in the A1 of the mouse, specifically layer 2/3, with no sensori-behavioural markers inherent in neurons and their connectivity, given that area of the brain, in that specific part of the cortical column, by cytoarchitecture, is populated by neurons with structural and perhaps functional similarities to a high degree. To the point, how do the authors square layer 2/3 neurons, characterised almost exclusively as feedforward neurons, with the inverse solution, which requires some form of feedback neurons to implement? Otherwise, without these, the authors' model demonstrates no physical system, except the general notion of variance in a population activity, thus inherent distinct groups, indicated by temporal dynamics in spiking activity.

Response: We agree with the reviewer's assessment that our forward/inverse models are not aimed at establishing a physical circuit model, but a statistical model that captures the functional dependencies among neuron in terms of their activity. As such, the links that are found between neurons may not be

interpreted as direct synaptic connections. *To address this comment, we have now added this explanation to the discussion (page 25, lines 556-560).*

However, the manuscript was executed accurately and appropriately within the confines of its design. The findings reported truly reflect the manuscript's objectives. And in that regard, the title, abstract, and author summary capture the content of the text. The main text is clear and comprehensible. Nevertheless, "Granger taxonomy" seems an over-reach, given that it fails to live up to the standard of structural qualification grounded in physical form, required of a generative model.

Response: We agree that our proposed taxonomy is "functional" and not "physical". As such, we have now changed the title of the paper as "Sensori-behaviorial **Functional** Taxonomy", with a similar phrasing used throughout the paper to avoid any unintentional over-reach.

Response to Reviewer 2's Comments

Reviewer #2: The authors present a framework to extract directional interactions between stimuli, their neural responses and corresponding behavior on two-photon calcium imaging data. This method relies on finding directed acyclic graphs, where nodes serve as neural ensembles active during two-photon imaging, and the directionality of information is determined using Granger causality (GC). This work extends a vast body of research to construct causal relationships and functional connectivity graphs across brain regions based on proxy signals for neural activity when electrophysiological data may be infeasible to obtain. This technique could thus also be relevant for the community developing tools for non-invasive brain imaging. I invite the authors to clarify the following before publication of this work.

Response: We thank the reviewer for their positive assessment of our work.

- As the work promises to be taxonomical, I want to get a sense of how this method would behave given representational drift (Schoonover et al. 2021) in encoding sensory information. It seems that we would need to run the GC analysis repeatedly over time to recover the taxonomical labels for neural ensembles which may be time consuming. Furthermore, it may be favorable for neurons to switch their labels based on the demands of the task. What is the temporal window over which a given Bayesian network topology remains stable?

Response: This is a good point. All analysis has been done within a trial, so we assume no drift occurs within a trial. Though, averaging across trials within an experimental session would only capture an average-sense taxonomy which could be confounded with representational drift. It is possible to extend our framework to a time-varying scheme (e.g., Rupasinghe et al. (2017)) to capture representational drift and account for it in attributing the G-taxonomy. *We have now added this point to the discussion (page 27, lines 606-614).*

- GC is highly sensitive to intrinsic oscillations and relies on separable signals. There is evidence that oscillatory brain state are detectable even in two-photon calcium signals (Tort-Colet et al. 2023), which implies that in the present study, the GC method could have spuriously linked periodic task stimuli with the activity of neural ensemble(s). How does the current method account for this effect? Similarly, it may be insightful to discuss the merits or limitations of GC-based approach vis-a-vis other manifold driven causality techniques like Convergent Cross Mapping which relax the constraints of separability.

Response: Here we did not observe any brain-wide oscillations within a trial. This is likely due to the fact that Tort-Colet (2023) used GCaMP6f while we used the much slower GCaMP6s. However, if such oscillations exist, as pointed out in the reference by the reviewer, GC can be severely confounded. *We have now added this to discussion and pointed out CCM as an alternative approach that could address scenarios in which a common oscillatory source is driving the network (page 26, lines 616-631).*

- In Figure 1, a first order kernel with additive noise is used to translate spikes into proxy GCaMP-fluorescence signal. Since this is a model of a synapse, would a multiplicative noise model be a better fit? This might better capture synaptic release probability.

Response: This a good point. This was done for the sake of simplicity and tractability of state-space inference. A multiplicative noise model would certainly be a better match to synaptic physiology but would make the inference quite complicated.

- In the toy model example (lines 208-213), the authors add a fixed variance white noise to the primary stimulus. As the GC method is also noise sensitive, it would be insightful to see at which noise levels does the analysis yield incorrect network topology. In other words, what minimum signal-to-noise ratio can this method reliably resolve in this simulated setup?

Response: This is a great suggestion for assessing the robustness of our method to stimulus representation mismatch. *We have now added this perturbational analysis to Fig. 3, in addition to other similar suggestions by Reviewer 3 (Fig. 3 on page 13, and page 15, lines 282-292):*

Signal-to-noise ratio (SNR) of the Primary Stimulus: Recall that in our generative model, we added a background zero-mean white noise with variance *0.1* to the primary stimulus, to account for mismatch in stimulus representation, whereas the estimator in the inverse model is not aware of such mismatch. This amounts to an SNR of *20* dB in the baseline comparison. To further assess the effect of this mismatch, we increased the noise and repeated the comparison for SNR levels of *10* and *5* dB, which are shown in Fig. 3B and C, respectively. While the performance of the 2P and two-stage PSTH methods degrades severely as SNR decrease, especially in terms of detecting Granger stimulus neurons, and thereby Granger intersection neurons, our proposed method exhibits more noise resilience and operates reliably for SNRs as low as *5* dB.

- The passive listening task presents a unique opportunity to probe the compulsive aspects of behavior independent of the stimulus. For instance, the trial-to-trial variability in neural responses is strongly associated with stereotypical behavior (e.g. whisker movement in mice) (Mussel et al. 2019). It would be insightful to include analysis for GB links in this experiment as well. This measure could, for instance, also be used to determine a better baseline of neural activity which accounts for these stimulus independent behaviors.

References

Schoonover, C.E., Ohashi, S.N., Axel, R. and Fink, A.J., 2021. Representational drift in primary olfactory cortex. Nature, 594(7864), pp.541-546.

Tort-Colet, N., Resta, F., Montagni, E., Pavone, F., Allegra Mascaro, A.L. and Destexhe, A., 2023. Assessing brain state and anesthesia level with two-photon calcium signals. Scientific Reports, 13(1), p.3183.

Musall, S., Kaufman, M.T., Juavinett, A.L., Gluf, S. and Churchland, A.K., 2019. Single-trial neural dynamics are dominated by richly varied movements. Nature neuroscience, 22(10), pp.1677-1686.

Response: This is a nice point. This dataset did not include any behavioral measurements, such as pupillometry or whisking, so it would not be possible to identify GB links. If we had pupillometry or whisking data, this would have been a great analysis to perform. However, *we added this to the discussion that in the absence of a task-relevant behavior, one can also measure other behavioral signals and analyze GB neurons to find a better baseline of neural activity (page 26, lines 577-581).*

Response to Reviewer 3's Comments

Reviewer #3: Reviewer background: My research covers computational neuroscience in general. I have experience developing analysis methods for calcium data as well as analyzing calcium data.

In this work the authors tackle a difficult problem: how can we infer useful information about the neuron activities underlying calcium recordings? There are many previous approaches to this, but the authors and my own knowledge these largely rely on several separate steps involving e.g. spike detection and then further analysis (although see some of my criticisms below). It is then also hard, even after e.g. a spike detection step, to figure out how the different neurons relate to each other and task parameters. In this paper the authors leverage and combine several techniques to go straight from calcium imaging responses to a sensori-behavioral taxonomy.

Overall, I really liked the approach and ideas. However, I saw several major issues and have several open questions that I would need answered or solved before being able to recommend publication. I am recommending major revisions (although really they are between minor and major), but also think that the paper could be better at home in a more methods oriented journal in its current form.

Response: We thank the reviewer for their positive assessment of our work.

Code review: the code seems to be available and complete, but I am not able to test it due to not using MATLAB. The readme looks extensive and useful. However, much of the code itself seems sparsely commented and I would request the authors rectify this.

Response: Thanks for taking the time to go over the codes and for this suggestion. *We have now improved the commenting in the revised codes uploaded to the DRUM repository, which are under administrative review by the DRUM staff and should be updated shortly. We have also enclosed a temporary link to the updated MATLAB codes in our revised submission (as a Data Review URL). Rest assured that we will make sure the DRUM repository is updated with the revised codes prior to the publication of the paper.*

Major concerns

- The authors claim that unlike previous works they several a lot of things simultaneously unlike previous studies (in particular, they skip any spike detection but go directly from calcium recordings to their analysis). However, this is not completely fair, as there several previous approaches that also take several analysis steps at once, just at a different point in the analysis pipeline. For example, approaches like cNMF [1] simultaneously detect cell locations, calcium traces, and spiking activities. Then, follow-up approaches are done on the spiking or calcium traces. From my understanding, the approach taken in this paper assumes cell locations and calcium traces are already provided, so also still skips some part of the analysis. Leading to the next point:

Response: This is a fair point. *We have now clarified in the discussion that the spatial locations of the cells need to be determined before our analysis can be run, which is not required by some of the existing methods such as cNMF (page 24, lines 532-536).*

- The paper insufficiently details what preprocessing is required of the data before their method is applied. I'm particularly worried about denoising and de-contamination of the cell signals. For example, neuropil contamination can seriously bias analysis methods [2], and could also explain some of the causality links they find in the experimental data. Neuripil contamination can come from overlapping cells in the imaging cone, as well as other surrounding tissue. What is more, Neuropil contamination can specifically be highly tuned to task parameters as well (see also [2]). How is such contamination either accounted for in pre-processing or in their analysis method itself? It is possible I misunderstood some of the method construction and that this is already taken into account.

Response: This is a good point. We had indeed performed neuropil contamination subtraction as a pre-processing step but did not explain it here and instead cited the references Rupasinge et al. (2021) and Francis et al. (2022) from which our experimental data was adopted. *We have now added a self-contained section to the Methods that describes the details of the pre-processing steps, including neuropil contamination subtraction (page 45, lines 977-989).*

- In figure 3A+B it seems that the spiking trace does not fully conform to the calcium trace. There are clear bumps in the calcium trace that do not conform to spikes. Perhaps this is the result of modelling something like the neuropil contamination I mention above? However, from the generative model (equation 1), and the fact that A there is diagonal, this does not seem to be the case (as well as how figure 1 illustrates the generative model).

Response: Thanks for this good catch. This was due to a plotting/coloring error in Adobe Illustrator. *We have now replotted Fig. 3A and B completely (now labeled Fig. 2B and D, respectively) and the issue regarding the correspondence of spikes and calcium concentrations is resolved. To make this correspondence clearer, we have now marked each spike on the calcium trace with a red dot (page 12, Fig. 2).*

- The paper uses quite a lot terminology and abbreviations which might be unfamiliar to much of the PLOS comp bio audience (at least to the single audience data point that is me!). This makes much of the text hard to follow. Consider for example the text below figure 5 on page 18. We GS and GB are used in abundance, but I had to cross reference a lot to other text to make sense of the text as a whole. Other examples are sentences like 'in the sense of granger' or 'in the sense of Shannon', which I found confusing. There are other similar examples throughout the text. I expect experimental neuroscientists to have similar issues as me. I recommend to both reduce the number of uses of GB, GS, GI terms in general, and to consider using written out terms through instead as well (e.g. just write 'Granger Behavior Neuron' everywhere, while also reducing over-use of the term). Finally, I think some of the terminology could be phrased more intuitively, or perhaps a box could be used to give a quick intro to granger-related terminology.

Response: We have now changed the instances of "in the sense of Shannon" to "in the sense of Shannon's ***mutual information***", and "in the sense of Granger" to "in the sense of Granger's ***temporal predictability***" to be explicit. Regarding the terminology table, we had intended for the bottom panel of Fig. 1 to serve such a purpose. While we agree that spelling out GS, GB and GI will make the text less confusing, it would result in excessive repetitions of the words "Granger" and "Neuron". *As a middle ground, we have now revised the sections of the text, e.g., description of Fig. 5 as pointed out by the reviewer (pages 21-23), to spell out some of these acronyms that are repeatedly used in a paragraph.* But, when they are used sparsely throughout a paragraph, we have chosen to retain them as acronyms.

- The authors first do a simulation study to proof their method can extract the right taxonomy, and then apply their method to real experimental data. For the simulation study I found that it was quite hard to parse how exactly the data was generated, and I recommend to improve this part of the text to make this clearer (perhaps a dedicated methods section after the generative model explanation?). Secondly, I think a more extensive and rigorous study is required to show that their method in general can extract correct taxonomies from realistic datasets. In particular, the considered networks are small and few, and match the method's underlying generative model perfectly. This could be improved by e.g. considering a larger set of sampled taxonomies as well as much larger networks (then subsampled), or even considering datasets generated with a less perfectly matching generative model. Can we from this also define under what conditions the method might fail?

Response: This is a great suggestion. *We have now significantly revised Fig. 3, to also address a similar comment by reviewer 2 by performing the suggested perturbational analyses (Fig. 3 on page 13, and page 15-16, lines 293-326):*

Spiking Model Mismatch: In our baseline model, we assumed a Bernoulli spiking model in both the generative and inverse models. In practice, however, these two spiking models could be different. We thus generated the data using a Poisson spiking model but used a Bernoulli spiking model for the inverse model, thus inducing a spiking model mismatch. The ROC comparisons are shown in Fig. 3-D. While our method is able to produce high hit rates and moderate false alarm rates under this scenario, the two benchmark methods are not capable of recovering any of the Granger stimulus and intersection neurons.

Network Sub-sampling: The baseline comparison corresponds the simulated activity of $N = 10$ neurons, which is a modest number compared to the typical number of neurons observed using two-photon imaging. In practice, the number of neurons used for network analysis is usually a small fraction of the observed neurons, due to the computational requirements of such analysis. This network sub-sampling procedure could thus confound the analysis results, as many of the key underlying processes remain latent. To assess the robustness of our proposed method to this condition, we simulated a network of $N = 50$ neurons, with a VAR model ensuring at most *8* source neurons are connected to each target neuron. Then, we assigned *10* Granger sensory, *10* Granger behavior, and *5* Granger intersection neurons in a similar fashion as in the case of *10* neurons in our baseline model. Finally, we randomly sub-sampled *10* networks of size $N = 10$ neurons out of *50*, and performed the ROC evaluation shown in Fig. 3-E. As expected, the GC detection performance of all methods degrades, due to the confounding effect of network sub-sampling. Remarkably, our method is able to recover both GS and GI neurons at a high hit rate and low false alarm rate, whereas the other two benchmarks detect none.

These perturbational analyses suggest that while more realistic experimental conditions would affect the performance of our proposed G-taxonomy, it exhibits a higher degree of robustness to said perturbations compared to the existing benchmarks.

For the experimental data, aside from the issues in the previous point with parsing the text, I was hoping for some more reflection on what we can learn from the taxonomy. It seems that many of the major conclusions are confirmations of previous results. I think the paper would benefit by making more explicit what was already known previously and what new we have learned (or could learn in principle, even if their current dataset was just not sufficient for example).

Response: Thanks for this suggestion. *We have now explicitly mentioned and elaborated on what are the points that match existing literature (e.g., Francis et al. (2022), Figs. 2, 4 and S3) on pages 20-22.*

- The experimental results in general lacks an explicit comparison with other previous methods. For example, the method mentioned a few times (II) could be used on the simulation or experimental data as well, and reported on in the figures (or more specific references could be made to previous literature, if the data is hard to come by or the analyses hard to do).

Response: Given that II is not easy to scale-up to time-varying stimuli and large networks, as we have now elaborated on in the Computational Complexity section, we cannot directly compare with II. *However, we have now made more specific references to existing literature that have used II on these data (e.g., Francis et al. (2022), Fig. 2) on page 22.*

- A few times it is mentioned experimental analysis results found are consistent with previous papers, without making a specific reference to where in the papers this is also shown, and in what way they are consistent. This can be easily rectified by being more specific, and I would also consider to make a dedicated figure showing some of these correspondences more directly (if possible given datasets available and so on).

Response: To address this comment, *we have now made more specific references to existing literature as mentioned above (pages 20-22).*

- In the discussion it is mentioned no larger analyses were done due to computational constraints. But one of the major advances over the previous method (II) is supposed to be lower complexity. This point I think deserved a dedicated results section (both in reporting in more detail about the computational cost and constraints, and a comparison with II). Secondly, a view on how the method could be extended to larger datasets in the future would be appreciated, as calcium imaging datasets increasingly use very large groups of neurons. Thirdly, as also mentioned above, the simulation study could be used to generate a larger dataset then sub-sampled.

Response: This is a great suggestion. *We have now added a new dedicated computational complexity analysis section to the methods that compares the computational and sample complexities of our method with that of II and PID (page 43, lines 918-946).* Now, we rigorously show that II and PID require super-exponential computational complexity in the number of neurons, whereas our approach only requires polynomial complexity. In regard to applying our method to larger datasets, please also see our response to the previous comment regarding the new simulation study with sub-sampling.

Minor comments and questions

- (very minor) The paper a few times uses sentences like 'we presented a carefully designed simulation' or 'by a careful combination of'. I find such terms vague and it should be up to the reader to judge if indeed the design was careful or not, not for the authors to claim. It's better to be immediately precise about how the design is done, or how the combination is done.

Response: Fair point. *We have now revised the language to be more precise.*

- The figure captions were hard to fully grasp sometimes. For example, figure 3C is for 10 network realizations but this is not mentioned in the caption.

Response: Good point. *We have now added this missing information to the captions (page 13).*

- I would combine figure 2 and figure 3 top row, and make figure 3 bottom row its own figure or combine it as well (since the top row is on specific examples like figure 2).

Response: This is a great suggestion which *we have now happily incorporated.* This also makes it easier to add the ROC curves of the new simulations to the new Figure 3 *(page 13)*.

- How the 10 different networks for 3C are generated is not clear, should be part of methods probably.

Response: *We have now clarified this in the Results section by adding a dedicated paragraph to random network realizations (page 14, lines 249-257).*

- The benchmark methods should be fully explained in the methods.

Response: We have now added a section to the methods that gives a more detailed description of the benchmark methods *(page 44, lines 947-963).*

- Question: I found it interesting to calculate specific average responses only for subsets of neurons according to classification. Is there any relationship with and benefit over methods like demixed PCA for this?

Response: This is a very nice observation that we didn't think of before. Our proposed G-taxonomy can also be thought of as a clustering methodology, which explicitly uses the functional sensori-behavioral relevance of the neurons' activity to categorize them, akin to methods such as demixed PCA. *We have now added this as a discussion point (page 25, lines 560-563).*

- Figure 1: very minor, but the ordering of GS, GI, and GB is not consistent between figure , text, and caption

Response: *Fixed*.

- Question: would terms like 'Stimulus linked neuron' perhaps be more intuitive than 'Granger stimulus neuron', even if it is less specific? Or does this not cover the outcome of the analysis enough.

Response: We also had a similar question in mind while writing the paper but eventually chose to keep the word "Granger" to highlight that our methodology is based on Granger's temporal predictability formalism.

Typo's and others

- line 318 - '*this* results'

Response: *Fixed*.

- line 76 'of methods such as' is a vague statement, better to just say what methods were used specifically

Response: *Fixed*.

- line 118 'the *i* neuron'

Response: *Fixed*.

- line 152+153: double use of 'here,....'

Response: *Fixed*.

- line 406 'the *to* neuron'

Response: *Fixed*.

[1] Pnevmatikakis, E. A., Soudry, D., Gao, Y., Machado, T. A., Merel, J., Pfau, D., ... & Paninski, L. (2016). Simultaneous denoising, deconvolution, and demixing of calcium imaging data. Neuron, 89(2), 285-299.

[2] Keemink, S. W., Lowe, S. C., Pakan, J. M., Dylda, E., Van Rossum, M. C., & Rochefort, N. L. (2018). FISSA: A neuropil decontamination toolbox for calcium imaging signals. Scientific reports, 8(1), 3493.

Response: *These new references are now added in relevant sections of the manuscript.*
