# Supplementary material for: Granger sensori-behavioral functional taxonomy of neuronal ensemble activity from two-photon calcium imaging data
Source: PLoS Comput Biol. 2026 Sep 30;22(9):e1014820. doi: 10.1371/journal.pcbi.1014820 (PMC13660992; doi:10.1371/journal.pcbi.1014820)
Supplement: Peer review history

# Peer review: Decision Letter 0

**DOI:** 10.1371/journal.pcbi.1014820.r001

**Authors:** Daniel Bush, Alain Nogaret

17 May 2026

Granger Sensori-Behavioral Taxonomy of Neuronal Ensemble Activity from Two-Photon Calcium Imaging Data

PLOS Computational Biology

Dear Dr. Babadi,

Thank you for submitting your manuscript to PLOS Computational Biology. After careful consideration, we feel that it has merit but does not fully meet PLOS Computational Biology's publication criteria as it currently stands. Therefore, we invite you to submit a revised version of the manuscript that addresses the points raised during the review process.

Please submit your revised manuscript by Jul 17 2026 11:59PM. If you will need more time than this to complete your revisions, please reply to this message or contact the journal office at ploscompbiol@plos.org. When you're ready to submit your revision, log on to https://www.editorialmanager.com/pcompbiol/ and select the 'Submissions Needing Revision' folder to locate your manuscript file.

Please include the following items when submitting your revised manuscript:

* A letter that responds to each point raised by the editor and reviewer(s). You should upload this letter as a separate file labeled 'Response to Reviewers'. This file does not need to include responses to formatting updates and technical items listed in the 'Journal Requirements' section below.

* A marked-up copy of your manuscript that highlights changes made to the original version. You should upload this as a separate file labeled 'Revised Manuscript with Track Changes'.

* An unmarked version of your revised paper without tracked changes. You should upload this as a separate file labeled 'Manuscript'.

As the corresponding author, your ORCID iD is verified in the submission system and will appear in the published article. PLOS supports the use of ORCID, and we encourage all coauthors to register for an ORCID iD and use it as well. Please encourage your coauthors to verify their ORCID iD within the submission system before final acceptance, as unverified ORCID iDs will not appear in the published article. Only the individual author can complete the verification step; PLOS staff cannot verify ORCID iDs on behalf of authors.

We look forward to receiving your revised manuscript.

Kind regards,

Alain Nogaret, PhD

Academic Editor

PLOS Computational Biology

Daniel Bush

Section Editor

PLOS Computational Biology

**Additional Editor Comments:**

The three reviewers are positive about the approach developed in the paper. However they have also made extensive comments and suggestions that need addressing for the paper to meet publication requirements.

**Journal Requirements:**

If the reviewer comments include a recommendation to cite specific previously published works, please review and evaluate these publications to determine whether they are relevant and should be cited. There is no requirement to cite these works unless the editor has indicated otherwise.

1) We ask that a manuscript source file is provided at Revision. Please upload your manuscript file as a .doc, .docx, .rtf or .tex. If you are providing a .tex file, please upload it under the item type u2018LaTeX Source Fileu2019 and leave your .pdf version as the item type u2018Manuscriptu2019.

2) Please upload all main figures as separate Figure files in .tif or .eps format. For more information about how to convert and format your figure files please see our guidelines:

https://journals.plos.org/ploscompbiol/s/figures

3) Some material included in your submission may be copyrighted. According to PLOS’s copyright policy, authors who use figures or other material (e.g., graphics, clipart, maps) from another author or copyright holder must demonstrate or obtain permission to publish this material under the Creative Commons Attribution 4.0 International (CC BY 4.0) License used by PLOS journals. Please closely review the details of PLOS’s copyright requirements here: PLOS Licenses and Copyright. If you need to request permissions from a copyright holder, you may use PLOS's Copyright Content Permission form.

Please respond directly to this email and provide any known details concerning your material's license terms and permissions required for reuse, even if you have not yet obtained copyright permissions or are unsure of your material's copyright compatibility. Once you have responded and addressed all other outstanding technical requirements, you may resubmit your manuscript within Editorial Manager.

Potential Copyright Issues:

i) Figure 1. Please confirm whether you drew the images / clip-art within the figure panels by hand. If you did not draw the images, please provide (a) a link to the source of the images or icons and their license / terms of use; or (b) written permission from the copyright holder to publish the images or icons under our CC BY 4.0 license. Alternatively, you may replace the images with open source alternatives. See these open source resources you may use to replace images / clip-art:

- https://commons.wikimedia.org

- https://openclipart.org/.

4) Please amend your detailed Financial Disclosure statement. This is published with the article. It must therefore be completed in full sentences and contain the exact wording you wish to be published.

1) State what role the funders took in the study. If the funders had no role in your study, please state: "The funders had no role in study design, data collection and analysis, decision to publish, or preparation of the manuscript."

2) If any authors received a salary from any of your funders, please state which authors and which funders..

If you did not receive any funding for this study, please simply state: u201cThe authors received no specific funding for this work.u201d

**Reviewers' comments:**

Reviewer's Responses to Questions

**Comments to the Authors:**

**Please note here if the review is uploaded as an attachment.**

Reviewer #1: The manuscript by Khostavi et al., entitled “Granger Sensori-Behavioural Taxonomy of Neuronal Ensemble Activity from Two-Photon Calcium Imaging Data”, immediately stands out as a well-thought-out and executed piece of work that improves our understanding of neuronal computation. It sets out to build and test a computational model that provides an all-in-one account of sensory processing. By all-in-one, they mean both the encoding and decoding of stimuli by neurons, among other things, which together constitute the neuronal response. Therefore, it is laudable that the authors took an approach that is contrary to the previous approach, which slices up neural activity in the study of one or the other, i.e., encoding or decoding of sensory information. With the latter approach, recent developments in statistical hypothesis testing have shown that type I error, thus false positives, abound.

The authors' model is a classification system that describes three categories of neurons related to sensory and behavioural information in neural ensemble activity, based on a correlational metric known as Granger formalism. To be able to infer information, in the spirit of existing frameworks like intersection information and partial information decomposition, Granger causality (GC) presents a robust parametric tool to identify directional effects and within multivariate point process data. To this end, the three categories of neurons were defined as Granger stimulus encoding (GS), Granger behavioural decoding (GB), and Granger Intersection (GI), guided by efficient statistical testing, control of false discovery rate, and effective estimation of network parameters.

To test the model, the authors sought to identify GS, GB, and GI in two-photon calcium imaging data. Further, the marked neurons were hypothesised to approximate neurons in the behaving mouse A1, with similar functions. The results showed a significant presence of GC neurons in both datasets.

While the authors presented good evidence to support the model, there remain significant points that allow for confidence in the utility of the model to be shaken, especially for experimenters as well as theoreticians. These points can be put into two folds: manifested or assumed properties of neurons ascribed to GC neurons and the physical connectivity of identified properties between GC neurons. By their nature, models are physical description of the constitution and mechanism of systems. Hence, the authors' model lacks a cortical structure and precise transduction of information between GC neurons, which the statistical framework, Granger formalism, was supposed to elucidate. For instance, GS, GB, and GI neurons were shown to characterise diverse groups of neurons in the A1 of the mouse, specifically layer 2/3, with no sensori-behavioural markers inherent in neurons and their connectivity, given that area of the brain, in that specific part of the cortical column, by cytoarchitecture, is populated by neurons with structural and perhaps functional similarities to a high degree. To the point, how do the authors square layer 2/3 neurons, characterised almost exclusively as feedforward neurons, with the inverse solution, which requires some form of feedback neurons to implement? Otherwise, without these, the authors' model demonstrates no physical system, except the general notion of variance in a population activity, thus inherent distinct groups, indicated by temporal dynamics in spiking activity.

However, the manuscript was executed accurately and appropriately within the confines of its design. The findings reported truly reflect the manuscript’s objectives. And in that regard, the title, abstract, and author summary capture the content of the text. The main text is clear and comprehensible. Nevertheless, “Granger taxonomy” seems an over-reach, given that it fails to live up to the standard of structural qualification grounded in physical form, required of a generative model.

Reviewer #2: The authors present a framework to extract directional interactions between stimuli, their neural responses and corresponding behavior on two-photon calcium imaging data. This method relies on finding directed acyclic graphs, where nodes serve as neural ensembles active during two-photon imaging, and the directionality of information is determined using Granger causality (GC). This work extends a vast body of research to construct causal relationships and functional connectivity graphs across brain regions based on proxy signals for neural activity when electrophysiological data may be infeasible to obtain. This technique could thus also be relevant for the community developing tools for non-invasive brain imaging. I invite the authors to clarify the following before publication of this work.

- As the work promises to be taxonomical, I want to get a sense of how this method would behave given representational drift (Schoonover et al. 2021) in encoding sensory information. It seems that we would need to run the GC analysis repeatedly over time to recover the taxonomical labels for neural ensembles which may be time consuming. Furthermore, it may be favorable for neurons to switch their labels based on the demands of the task. What is the temporal window over which a given Bayesian network topology remains stable?

- GC is highly sensitive to intrinsic oscillations and relies on separable signals. There is evidence that oscillatory brain state are detectable even in two-photon calcium signals (Tort-Colet et al. 2023), which implies that in the present study, the GC method could have spuriously linked periodic task stimuli with the activity of neural ensemble(s). How does the current method account for this effect? Similarly, it may be insightful to discuss the merits or limitations of GC-based approach vis-a-vis other manifold driven causality techniques like Convergent Cross Mapping which relax the constraints of separability.

- In Figure 1, a first order kernel with additive noise is used to translate spikes into proxy GCaMP-fluorescence signal. Since this is a model of a synapse, would a multiplicative noise model be a better fit? This might better capture synaptic release probability.

- In the toy model example (lines 208-213), the authors add a fixed variance white noise to the primary stimulus. As the GC method is also noise sensitive, it would be insightful to see at which noise levels does the analysis yield incorrect network topology. In other words, what minimum signal-to-noise ratio can this method reliably resolve in this simulated setup?

- The passive listening task presents a unique opportunity to probe the compulsive aspects of behavior independent of the stimulus. For instance, the trial-to-trial variability in neural responses is strongly associated with stereotypical behavior (e.g. whisker movement in mice) (Mussel et al. 2019). It would be insightful to include analysis for GB links in this experiment as well. This measure could, for instance, also be used to determine a better baseline of neural activity which accounts for these stimulus independent behaviors.

References

Schoonover, C.E., Ohashi, S.N., Axel, R. and Fink, A.J., 2021. Representational drift in primary olfactory cortex. Nature, 594(7864), pp.541-546.

Tort-Colet, N., Resta, F., Montagni, E., Pavone, F., Allegra Mascaro, A.L. and Destexhe, A., 2023. Assessing brain state and anesthesia level with two-photon calcium signals. Scientific Reports, 13(1), p.3183.

Musall, S., Kaufman, M.T., Juavinett, A.L., Gluf, S. and Churchland, A.K., 2019. Single-trial neural dynamics are dominated by richly varied movements. Nature neuroscience, 22(10), pp.1677-1686.

Reviewer #3: Reviewer background: My research covers computational neuroscience in general. I have experience developing analysis methods for calcium data as well as analyzing calcium data.

In this work the authors tackle a difficult problem: how can we infer useful information about the neuron activities underlying calcium recordings? There are many previous approaches to this, but the authors and my own knowledge these largely rely on several separate steps involving e.g. spike detection and then further analysis (although see some of my criticisms below). It is then also hard, even after e.g. a spike detection step, to figure out how the different neurons relate to each other and task parameters. In this paper the authors leverage and combine several techniques to go straight from calcium imaging responses to a sensori-behavioral taxonomy.

Overall, I really liked the approach and ideas. However, I saw several major issues and have several open questions that I would need answered or solved before being able to recommend publication. I am recommending major revisions (although really they are between minor and major), but also think that the paper could be better at home in a more methods oriented journal in its current form.

Code review: the code seems to be available and complete, but I am not able to test it due to not using MATLAB. The readme looks extensive and useful. However, much of the code itself seems sparsely commented and I would request the authors rectify this.

Major concerns

- The authors claim that unlike previous works they several a lot of things simultaneously unlike previous studies (in particular, they skip any spike detection but go directly from calcium recordings to their analysis). However, this is not completely fair, as there several previous approaches that also take several analysis steps at once, just at a different point in the analysis pipeline. For example, approaches like cNMF [1] simultaneously detect cell locations, calcium traces, and spiking activities. Then, follow-up approaches are done on the spiking or calcium traces. From my understanding, the approach taken in this paper assumes cell locations and calcium traces are already provided, so also still skips some part of the analysis. Leading to the next point:

- The paper insufficiently details what preprocessing is required of the data before their method is applied. I'm particularly worried about denoising and de-contamination of the cell signals. For example, neuropil contamination can seriously bias analysis methods [2], and could also explain some of the causality links they find in the experimental data. Neuripil contamination can come from overlapping cells in the imaging cone, as well as other surrounding tissue. What is more, Neuropil contamination can specifically be highly tuned to task parameters as well (see also [2]). How is such contamination either accounted for in pre-processing or in their analysis method itself? It is possible I misunderstood some of the method construction and that this is already taken into account.

- In figure 3A+B it seems that the spiking trace does not fully conform to the calcium trace. There are clear bumps in the calcium trace that do not conform to spikes. Perhaps this is the result of modelling something like the neuropil contamination I mention above? However, from the generative model (equation 1), and the fact that A there is diagonal, this does not seem to be the case (as well as how figure 1 illustrates the generative model).

- The paper uses quite a lot terminology and abbreviations which might be unfamiliar to much of the PLOS comp bio audience (at least to the single audience data point that is me!). This makes much of the text hard to follow. Consider for example the text below figure 5 on page 18. We GS and GB are used in abundance, but I had to cross reference a lot to other text to make sense of the text as a whole. Other examples are sentences like 'in the sense of granger' or 'in the sense of Shannon', which I found confusing. There are other similar examples throughout the text. I expect experimental neuroscientists to have similar issues as me. I recommend to both reduce the number of uses of GB, GS, GI terms in general, and to consider using written out terms through instead as well (e.g. just write 'Granger Behavior Neuron' everywhere, while also reducing over-use of the term). Finally, I think some of the terminology could be phrased more intuitively, or perhaps a box could be used to give a quick intro to granger-related terminology.

- The authors first do a simulation study to proof their method can extract the right taxonomy, and then apply their method to real experimental data. For the simulation study I found that it was quite hard to parse how exactly the data was generated, and I recommend to improve this part of the text to make this clearer (perhaps a dedicated methods section after the generative model explanation?). Secondly, I think a more extensive and rigorous study is required to show that their method in general can extract correct taxonomies from realistic datasets. In particular, the considered networks are small and few, and match the method's underlying generative model perfectly. This could be improved by e.g. considering a larger set of sampled taxonomies as well as much larger networks (then subsampled), or even considering datasets generated with a less perfectly matching generative model. Can we from this also define under what conditions the method might fail?

For the experimental data, aside from the issues in the previous point with parsing the text, I was hoping for some more reflection on what we can learn from the taxonomy. It seems that many of the major conclusions are confirmations of previous results. I think the paper would benefit by making more explicit what was already known previously and what new we have learned (or could learn in principle, even if their current dataset was just not sufficient for example).

- The experimental results in general lacks an explicit comparison with other previous methods. For example, the method mentioned a few times (II) could be used on the simulation or experimental data as well, and reported on in the figures (or more specific references could be made to previous literature, if the data is hard to come by or the analyses hard to do).

- A few times it is mentioned experimental analysis results found are consistent with previous papers, without making a specific reference to where in the papers this is also shown, and in what way they are consistent. This can be easily rectified by being more specific, and I would also consider to make a dedicated figure showing some of these correspondences more directly (if possible given datasets available and so on).

- In the discussion it is mentioned no larger analyses were done due to computational constraints. But one of the major advances over the previous method (II) is supposed to be lower complexity. This point I think deserved a dedicated results section (both in reporting in more detail about the computational cost and constraints, and a comparison with II). Secondly, a view on how the method could be extended to larger datasets in the future would be appreciated, as calcium imaging datasets increasingly use very large groups of neurons. Thirdly, as also mentioned above, the simulation study could be used to generate a larger dataset then sub-sampled.

Minor comments and questions

- (very minor) The paper a few times uses sentences like 'we presented a carefully designed simulation' or 'by a careful combination of'. I find such terms vague and it should be up to the reader to judge if indeed the design was careful or not, not for the authors to claim. It's better to be immediately precise about how the design is done, or how the combination is done.

- The figure captions were hard to fully grasp sometimes. For example, figure 3C is for 10 network realizations but this is not mentioned in the caption.

- I would combine figure 2 and figure 3 top row, and make figure 3 bottom row its own figure or combine it as well (since the top row is on specific examples like figure 2).

- How the 10 different networks for 3C are generated is not clear, should be part of methods probably.

- The benchmark methods should be fully explained in the methods.

- Question: I found it interesting to calculate specific average responses only for subsets of neurons according to classification. Is there any relationship with and benefit over methods like demixed PCA for this?

- Figure 1: very minor, but the ordering of GS, GI, and GB is not consistent between figure , text, and caption

- Question: would terms like 'Stimulus linked neuron' perhaps be more intuitive than 'Granger stimulus neuron', even if it is less specific? Or does this not cover the outcome of the analysis enough.

Typo's and others

- line 318 - '*this* results'

- line 76 'of methods such as' is a vague statement, better to just say what methods were used specifically

- line 118 'the *i* neuron'

- line 152+153: double use of 'here,....'

- line 406 'the *to* neuron'

[1] Pnevmatikakis, E. A., Soudry, D., Gao, Y., Machado, T. A., Merel, J., Pfau, D., ... & Paninski, L. (2016). Simultaneous denoising, deconvolution, and demixing of calcium imaging data. Neuron, 89(2), 285-299.

[2] Keemink, S. W., Lowe, S. C., Pakan, J. M., Dylda, E., Van Rossum, M. C., & Rochefort, N. L. (2018). FISSA: A neuropil decontamination toolbox for calcium imaging signals. Scientific reports, 8(1), 3493.

**********

**Have the authors made all data and (if applicable) computational code underlying the findings in their manuscript fully available?**

The PLOS Data policy requires authors to make all data and code underlying the findings described in their manuscript fully available without restriction, with rare exception (please refer to the Data Availability Statement in the manuscript PDF file). The data and code should be provided as part of the manuscript or its supporting information, or deposited to a public repository. For example, in addition to summary statistics, the data points behind means, medians and variance measures should be available. If there are restrictions on publicly sharing data or code —e.g. participant privacy or use of data from a third party—those must be specified.

Reviewer #1: Yes

Reviewer #2: Yes

Reviewer #3: Yes

**********

PLOS authors have the option to publish the peer review history of their article (what does this mean?). If published, this will include your full peer review and any attached files.

If you choose “no”, your identity will remain anonymous but your review may still be made public.

**Do you want your identity to be public for this peer review?**  For information about this choice, including consent withdrawal, please see our Privacy Policy.

Reviewer #1: **Yes:**  Jolly Kanjua

Reviewer #2: No

Reviewer #3: No

**Figure resubmission:**

After uploading your figures to PLOS’s NAAS tool - https://ngplosjournals.pagemajik.ai/artanalysis, NAAS will process the files provided and display the results in the "Uploaded Files" section of the page as the processing is complete. If the uploaded figures meet our requirements (or NAAS is able to fix the files to meet our requirements), the figure will be marked as "fixed" above. If NAAS is unable to fix the files, a red "failed" label will appear above. When NAAS has confirmed that the figure files meet our requirements, please download the file via the download option, and include these NAAS processed figure files when submitting your revised manuscript.

**Reproducibility:**

To enhance the reproducibility of your results, we recommend that authors of applicable studies deposit laboratory protocols in protocols.io, where a protocol can be assigned its own identifier (DOI) such that it can be cited independently in the future. Additionally, PLOS ONE offers an option to publish peer-reviewed clinical study protocols. Read more information on sharing protocols at https://plos.org/protocols?utm_medium=editorial-email&utm_source=authorletters&utm_campaign=protocols

# Author comment: Author response to Decision Letter 1

**DOI:** 10.1371/journal.pcbi.1014820.r002

17 Aug 2026

Attachment Submitted filename: Granger_Taxonomy_PLoS_Response.pdf

# Peer review: Decision Letter 1

**DOI:** 10.1371/journal.pcbi.1014820.r003

**Authors:** Daniel Bush, Alain Nogaret

16 Sep 2026

Dear Prof. Babadi,

We are pleased to inform you that your manuscript 'Granger Sensori-Behavioral Functional Taxonomy of Neuronal Ensemble Activity from Two-Photon Calcium Imaging Data' has been provisionally accepted for publication in PLOS Computational Biology.

Before your manuscript can be formally accepted you will need to complete some formatting changes, which you will receive in a follow up email. A member of our team will be in touch with a set of requests.

Please note that your manuscript will not be scheduled for publication until you have made the required changes, so a swift response is appreciated.

IMPORTANT: The editorial review process is now complete. PLOS will only permit corrections to spelling, formatting or significant scientific errors from this point onwards. Requests for major changes, or any which affect the scientific understanding of your work, will cause delays to the publication date of your manuscript.

Should you, your institution's press office or the journal office choose to press release your paper, you will automatically be opted out of early publication. We ask that you notify us now if you or your institution is planning to press release the article. All press must be co-ordinated with PLOS.

Thank you again for supporting Open Access publishing; we are looking forward to publishing your work in PLOS Computational Biology.

Best regards,

Alain Nogaret, PhD

Academic Editor

PLOS Computational Biology

Daniel Bush

Section Editor

PLOS Computational Biology

***********************************************************

The manuscript revision and responses address all major comments raised by reviewers. I am pleased to recommend publication.

Reviewer's Responses to Questions

**Comments to the Authors:**

**Please note here if the review is uploaded as an attachment.**

Reviewer #3: Many thanks for the review response and new results. Especially the new perturbation experiments (noise/model mismatch/subsampling) convinced to advice full acceptance, except for one final suggestion (which I don't need to be consulted on):

The results now clearly show that under sub-optimal conditions the method still works reasonably well, and especially better than the baseline methods. In addition, the new section on computational complexity clarifies how hard it might be to run the method. However, I think the text slightly oversells the results as being just overall good. I think it would still be very helpful for readers that might want to use the method to clearly understand the failure modes: what degree of subsampling/noise/etc. is still ok? What kind of datasets is the method good for? How many neurons can we feasibly apply this to?

**********

**Have the authors made all data and (if applicable) computational code underlying the findings in their manuscript fully available?**

The PLOS Data policy requires authors to make all data and code underlying the findings described in their manuscript fully available without restriction, with rare exception (please refer to the Data Availability Statement in the manuscript PDF file). The data and code should be provided as part of the manuscript or its supporting information, or deposited to a public repository. For example, in addition to summary statistics, the data points behind means, medians and variance measures should be available. If there are restrictions on publicly sharing data or code —e.g. participant privacy or use of data from a third party—those must be specified.

Reviewer #3: Yes

**********

PLOS authors have the option to publish the peer review history of their article (what does this mean?). If published, this will include your full peer review and any attached files.

If you choose “no”, your identity will remain anonymous but your review may still be made public.

**Do you want your identity to be public for this peer review?**  For information about this choice, including consent withdrawal, please see our Privacy Policy.

Reviewer #3: No

# Editor report: Acceptance letter

**DOI:** 10.1371/journal.pcbi.1014820.r004

**Authors:** Daniel Bush, Alain Nogaret

PCOMPBIOL-D-26-00488R1

Granger Sensori-Behavioral Functional Taxonomy of Neuronal Ensemble Activity from Two-Photon Calcium Imaging Data

Dear Dr Babadi,

I am pleased to inform you that your manuscript has been formally accepted for publication in PLOS Computational Biology. Your manuscript is now with our production department and you will be notified of the publication date in due course.

The corresponding author will soon be receiving a typeset proof for review, to ensure errors have not been introduced during production. Please review the PDF proof of your manuscript carefully, as this is the last chance to correct any errors. Please note that major changes, or those which affect the scientific understanding of the work, will likely cause delays to the publication date of your manuscript.

Soon after your final files are uploaded, unless you have opted out, the early version of your manuscript will be published online. The date of the early version will be your article's publication date. The final article will be published to the same URL, and all versions of the paper will be accessible to readers.

For Research, Software, and Methods articles, you will receive an invoice from PLOS for your publication fee after your manuscript has reached the completed accept phase. If you receive an email requesting payment before acceptance or for any other service, this may be a phishing scheme. Learn how to identify phishing emails and protect your accounts at https://explore.plos.org/phishing.

Thank you again for supporting PLOS Computational Biology and open-access publishing. We are looking forward to publishing your work!

With kind regards,

Sharmila Kamatchi

PLOS Computational Biology | Carlyle House, Carlyle Road, Cambridge CB4 3DN | United Kingdom ploscompbiol@plos.org | Phone +44 (0) 1223-442824 | ploscompbiol.org | @PLOSCompBiol
